# New Drugs, Appliances, and Things Medical

**Published:** 1890-03-08

**Authors:** 


### NEW DRUGS, APPLIANCES, AND THINGS MEDICAL.

[All preparations, appliances, novelties, etc., of which a notice is desired, should be sent to The Editor, The Lodge, Porchester Square, W.]

### ARTIFICIAL LIMBS.

Messrs. Ferris.

From the earliest times man has endeavoured to supply the loss of his limbs by artificial substitutes. Within the last year or two a bronze and wooden leg was found in a grave, of which the date, whether of Roman or earlier time, was disputed. We believe that something very similar has been found in Egypt—that cradle of early civilisation. Coming to later times, who has not read of the timber-toed Crowdero in Hudibras, or of the remarkable mechanical leg which when started would not stop, so that when last seen it had merely the skeleton of its miserable owner attached to it? The wooden legs of older England, in fact, and in fiction are innumerable, testifying in dotted accentuation to the centuries of warfare through which we have passed. We were led to make these preliminary remarks through a visit we paid a short time since to Messrs. J. and G. Ferris' rooms in Great Russell Street, in order to examine the artificial limbs made by them. We were greatly pleased with what we saw. The firm, by combining a number of ingenious inventions, has succeeded in making some of the most useful imbs we have inspected. The chief difficulty always has been in the leg. Beginning with the knee joint: Here we have a tendency to too great stiffness, so that the wearer, when he sits down, has to smite himself over the shin in order to bend his knee, or if the knee is too loose every step is completed by a blow, caused by the hinges of the knee coming together too quickly. Ferris obviates this by making the bearings cone pattern, and so arranging them that as they wear, they can be tightened up as required. The blow is prevented by a most ingenious cord arrangement in the back of the joint, so that as flexion is completed the pressure is gradually eased off. In the next joint, the ankle, still greater ingenuity has been displayed, and we can safely say that the nearest approach to nature has been obtained. There is a double joint, so that lateral as well as anteroposterior movement is allowed. This permits of the wearer going over the roughest ground.

The leg has also a hinge joint in the metatarso-phalangeal region, the spring of which, instead of going up to the calf or knee, is *in situ*, and so less complicated.

The mechanical arms are equally ingenious and well made. Both arms and legs are beautifully modelled, and Messrs. Ferris very wisely state that if the patient cannot attend to have the limb fitted and shaped, they always desire to have a plaster cast of the stump, so as to make the work exact.

We were much struck with the lightness of the limbs, and also their appearance. On inquiry we found that the limb is covered with wet hide, which, when dry, shrinks on to the wood tightly. This enables the makers to hollow out the wood to a much greater extent than before, and, consequently, makes the limb much lighter. It also allows of a coat of flesh-coloured enamel being applied uniformly, thus making the limb very seemly, and, what is of very great importance, enabling its owner to keep it absolutely clean by washing.

The firm also make an ingenious clump boot, by which the clump is put *inside* instead of outside the boot. Whilst we were examining the various limbs, a gentleman came into the room, and until Mr. Ferris drew our attention to him, we did not notice any peculiarity in his walk. However, he turned out to be a client who had lost his leg just below the knee. We asked him to put his heels together, and then stoop to pick a handkerchief off the floor. This he did with great ease. We need hardly say that the test is a severe one. He told us that he had walked as much as twelve miles in a day, could ride a horse or tricycle, and that he was enabled to follow his profession, that of an architect, as well as ever he had done before he lost his limb. Messrs. Ferris have taken three gold medals, 13 silver medals, and two highest awards. This is sufficient evidence of the quality of their inventions.

### PARAFFINUM MOLLE.

Since the introduction by the Chesebrough Manufacturing Company of the paraffin, which goes by their name of "Vaseline," a number of substitutes have appeared in the markets, and recently we were asked the question whether the soft paraffins or petroleum jellies made, or rather purified by chemical means, can be distinguished from those prepared by filtration under pressure. We have consulted many books and some of the most practical men on the subject. It seems that paraffinum molle is manufactured from the residues of the distillation of the more volatile petroleums. The natural petroleum oil is fractionally distilled, and the products are various. First comes a most volatile substance, which, when condensed under cold and pressure, is used like ether for freezing purposes. Second, the benzoline type of fluids distil over, these are used to dissolve india-rubber, fats, and oils, and for cleaning purposes. Third, the series of lamp oils of varying volatility. Fourth, an oil which when mixed with a proportion of animal or vegetable oil makes one of the best lubricating substances known. This, with the "vaselines," has a peculiar colour, hence one of its names, "blue oil." It is due to its florescence. The residues remaining form the paraffinum molle type of substances. These may be purified either by pressure, filtration, or by treatment with sulphuric acid, followed by soda solution and final purification with ammonia. We suppose the question put to us has regard to these two processes. Of course, if any of the salts of soda or ammonia, or even free acid, were to remain, we could understand that they would have a deleterious effect on the skin when used as ointments, but the detection of these impurities is so easy that there is no practical difficulty in finding them when present. Personally, we prefer the filtration to the chemical process. It is as well to note here that some people have an idiosyncracy with regard to the use of paraffin as ointment. They develop very quickly erythemata and eczema. We were also told that a good deal of mixed paraffins are sold as vaselines, that is, mixtures of the p. durum and p. molle. "Bloxam's Chemistry," 1888, states vaselines to be the paraffins distilling above 200 deg. C. Squire's "Companion to the British Pharmacopœia" says p. molle should show absence of added fatty matter, animal or vegetable, resin, or easily carbonized organic impurities, and gives the tests for these. The tests for free sulphuric acid, sodic and ammonia sulphates will be found in any text-book of chemistry. We may remark that in addition to the ordinary yellow vaselines there is a white variety sold; this is said to be made by filtering the yellow forms through animal charcoal. Again there are very useful forms of paraffinum molle in more liquid condition; we believe that the Dee Oil Company have a good specimen of this in the market. The great advantage the paraffins have over natural fats, animal and vegetable, is their non-liability to oxidation and change, so that the preparations made with them do not turn rancid. The chief disadvantage, omitting idiosyncrasy, of the more solid forms is the difficulty of absorption by the skin of the active ingredients of the preparations in which they occur. This may be overcome to some extent by more prolonged friction. In conclusion, we give one final hint with regard to the softer paraffin ointments containing heavy ingredients—do not let them become liquid by standing near the fire or otherwise, because the heavy materials are liable to sink to the bottom, and so the last portions used are apt to contain most of the active drug, and thus damage may be caused.

### DANIELSON'S CHARTS.

We have also received some charts and clinical figures from Messrs. Danielson and Co., which are of the greatest value. The "Perfect" clinical chart which can be had at 3s. the 100, fully deserves its name, and the forms of instruction for nurses are good. The clinical figures are outlines of the different part of the body on which can be marked the deformities or disease of any patient; these are more for students. Private nurses will, however, find them useful, and they will find the charts quite the best to be obtained.

